# Integrating gene and protein expression data with genome-scale metabolic networks to infer functional pathways

**DOI:** 10.1186/1752-0509-7-134

**Published:** 2013-12-08

**Authors:** Jon Pey, Kaspar Valgepea, Angel Rubio, John E Beasley, Francisco J Planes

**Affiliations:** 1 CEIT and TECNUN, University of Navarra, Manuel de Lardizabal 15, 20018 San Sebastian, Spain; 2 Department of Chemistry, Tallinn University of Technology, Akadeemia tee 15, 12618 Tallinn, Estonia; 3 Competence Centre of Food and Fermentation Technologies, Akadeemia tee 15a, 12618 Tallinn, Estonia; 4 Mathematical Sciences, Brunel University, John Crank 505, Kingston Lane, Uxbridge UB8 3PH, UK

**Keywords:** Acetate overflow, Gene expression, Proteomics, Systems biology, Metabolic pathways analysis, Mixed-integer linear programming

## Abstract

**Background:**

The study of cellular metabolism in the context of high-throughput -omics data has allowed us to decipher novel mechanisms of importance in biotechnology and health. To continue with this progress, it is essential to efficiently integrate experimental data into metabolic modeling.

**Results:**

We present here an *in-silico* framework to infer relevant metabolic pathways for a particular phenotype under study based on its gene/protein expression data. This framework is based on the Carbon Flux Path (CFP) approach, a mixed-integer linear program that expands classical path finding techniques by considering additional biophysical constraints. In particular, the objective function of the CFP approach is amended to account for gene/protein expression data and influence obtained paths. This approach is termed integrative Carbon Flux Path (iCFP). We show that gene/protein expression data also influences the stoichiometric balancing of CFPs, which provides a more accurate picture of active metabolic pathways. This is illustrated in both a theoretical and real scenario. Finally, we apply this approach to find novel pathways relevant in the regulation of acetate overflow metabolism in *Escherichia coli*. As a result, several targets which could be relevant for better understanding of the phenomenon leading to impaired acetate overflow are proposed.

**Conclusions:**

A novel mathematical framework that determines functional pathways based on gene/protein expression data is presented and validated. We show that our approach is able to provide new insights into complex biological scenarios such as acetate overflow in *Escherichia coli*.

## Background

Systems biology models biological processes at different hierarchical levels, ranging from genetic mechanisms to metabolic events as well as associated interactions and synergies. This “systems” perspective has allowed us to go beyond classical biology and chemistry, obtaining valuable insights regarding different fields: medicine [1,2], pharmacology research [3-5] and biotechnology [6] amongst others. The rapid growth of systems biology has been possible thanks to the advent of high-throughput -omics data [7] and efforts in the scientific community to develop mathematical and computational methods that integrate such data [8-11]. In order to continue with this pace of discoveries, extending and improving these integrative tools is a major challenge in systems biology.

In particular, the work presented here is focused on the metabolic layer. Metabolism has been intensively studied for the last decade due to its close relationship with cellular phenotype and, in consequence, with several diseases and biotechnological processes. The study of metabolism from a systems biology viewpoint has been accelerated by the publication of several genome-scale networks relating metabolites, enzymes and genes [12-14]. As discussed above, utilizing experimental data is essential to extract relevant biological conclusions regarding the particular scenario being modeled. In this light, three main families of experimental techniques provide a closer picture of cell metabolism: metabolomics [15], stable isotope labeling [16] and gene/protein expression measurements [17,18].

Gene expression can be quantified at the transcriptome level using DNA microarray technology [19], which provides a vast amount of information due to the low cost of measuring thousands of mRNAs simultaneously. Despite this vast amount of information, the number of relevant conclusions extracted from the direct analysis of these experiments has not met expectations [20]. This highlights the necessity for novel modeling frameworks accounting for gene and protein expression data [21]. In this context, pathway analysis tools arise as powerful alternatives [22]. Their application has already generated relevant insights, for example in health [23,24] or in biotechnology [25]. Complementary to transcriptome data, proteomics provides genome-wide information as to protein abundances, which is closer to the metabolic phenotype compared to gene expression data, by avoiding the intermediate regulatory mechanisms between mRNA expression and final protein production. However, the number of measured proteins is below the number of measured mRNAs, despite the recent rapid developments of proteomics [26]. In this article, we present a novel mathematical methodology integrating gene/protein expression data into metabolic networks with the objective of finding key pathways for the particular phenotype under study.

Pathway analysis tools can be classified into two general groups depending on how they are obtained, namely via manual curation [27,28] or via network-based techniques. In this second group, the use of path finding techniques has been extensive [29,30]. Whilst computationally efficient, these methods do not take into account reaction stoichiometry, which may lead to results that are not meaningful, as recognized in [31]. For this reason much effort is being carried out to explore other pathway concepts that consider mass-balances. One of the most important pathway concepts is the Elementary Flux Mode (EFM) [32]. In a pioneering work, Schwartz and co-workers [33] assign a probability to a set of EFMs calculated from KEGG maps [34] based on gene expression data. Recently, Rezola *et al.,*[35] has extended this work for genome-scale metabolic networks and determine a set of characteristic EFMs for different human healthy tissues based on gene expression data. Similarly to EFMs, [36] presented the concept of Elementary Flux Patterns. Recently, several biological insights have been obtained when gene expression data was projected into Elementary Flux Patterns [37]. In this article, we present the first framework mapping gene expression data into Carbon Flux Paths (CFPs) [38]. This approach introduces reaction stoichiometry into classical path finding techniques via Mixed-Integer Linear Programming (MILP). In particular, metabolites (nodes) are joined via reactions (arcs) representing effective carbon exchange. In addition, the obtained path is able to operate in sustained steady-state [38]. The versatility of CFP has been recently exploited to detect key enzymes whose malfunction is responsible for changes in metabolomic data [39].

The vast majority of approaches project gene expression data into a pre-calculated template containing a large number of pathways, regardless of the actual physiological scenario [28,33,40]. The inherent plasticity of the metabolic network may require pre-calculating a large number of pathways to guarantee that any biological scenario is properly represented. To avoid this limitation, other approaches calculate a particular set of pathways for each gene expression data set [41]. Our approach is in consonance with the discussion presented in [42], which suggests calculating specific pathways by weighting each reaction in terms of the corresponding enzyme expression. We directly find the most representative pathways in each scenario and enumerate top-ranked paths according to an objective function based on actual gene expression/protein data.

Finally, we apply our new approach to examine key pathways and find novel metabolic pathways involved in the regulation of acetate overflow metabolism in *E. coli*[43]. Acetate overflow, more precisely excretion of acetic acid, exists under aerobic *E. coli* growth on glucose at high specific growth rates [43,44] and severely inhibits growth and diverts carbon from biomass or target product synthesis [45,46]. Several hypotheses have been postulated for causing overflow metabolism of acetate, mainly involving imbalance between glucose uptake and TCA cycle or energy and biomass generation throughput [44,47]. Recently, Valgepea *et al.* 2010 [43] proposed a new regulation mechanism of acetate overflow being triggered by carbon catabolite repression of *acetyl-CoA synthetase* and subsequent disruption of the *phosphotransacetylase-acetyl-CoA synthetase* (PTA-ACS) node. Despite decades of study, the regulation mechanisms and all pathways involved have not been unequivocally determined making this metabolic phenomenon a very attractive one to study and test with the new mathematical modeling method developed in the current work.

## Results and discussion

With the CFP method [38] as a starting point, we aim to find relevant paths for a particular phenotype under study based on gene or protein expression data. As detailed in Pey *et al.*[38], the calculation of CFP is based on MILP, which allows us to (i) ensure that the obtained path can operate at sustained steady-state; (ii) force effective carbon exchange in each intermediate step; (iii) enumerate paths in increasing path length order. Here, we amend the CFP enumeration procedure regarding the gene and protein expression data. In addition, since CFPs take into account off-path reactions (not just fluxes for reactions involved in the path), gene expression data also guides the balancing of the path. Further methodological details can be found in the Methods section.

We first classify each metabolic reaction as Highly/Over expressed, Lowly/Down expressed or Medium expressed/Invariant based on gene and protein data sets, as done in [48]. For this, the set of highly/over expressed or lowly/down expressed genes/proteins is first determined. Several strategies can be adopted to perform this analysis [49]. Hereinafter, we apply the Boolean rules relating genes/proteins and reactions included in different metabolic network reconstructions [50,51], which finally leads to the definition of the set *H* of highly/over expressed reactions, the set *L* of lowly/down expressed reactions and the set *M* = *R*-*H*-*L* of medium expressed/invariant reactions (where *R* is the complete set of reactions). Further details as to how we classified reactions based on gene and protein expression data can be found in the Methods section.

Our model is constrained to have non-zero flux between the source and target metabolites forcing an additional set of reactions to be active so as to balance the path. Given this constraint, our optimization model is a three-stage minimization: firstly, minimize the total flux associated with reactions in *L*; then minimize the total flux associated with reactions in *M*; then minimize the length of the flux path from source to target metabolite. Note here how our model integrates gene expression data (as represented by the reaction sets *H*, *M* and *L*) via the objective functions in our three-stage minimization. This allows gene expression data to ***influence*** the flux scenario ***without directly constraining it***. In particular, we guide the flux through the reactions in *H* as a result of minimizing flux in *L* and *M*. Note that the third-stage optimization is a path length minimization step, as is common in the literature [52]. Conclusively, we call our approach ***iCFP (Integrated gene and protein expression Carbon Flux Paths)***.

We first highlight the inherent advantages of the iCFP approach with respect to existing approaches by means of a theoretical example. Afterwards, this theoretical example is reinforced in a more realistic scenario, namely in degradation of L-Alanine during biofilm formation of a genetically modified strain of *E. coli*. Finally, we apply iCFP for finding relevant metabolic pathways involved in acetate overflow metabolism in *E. coli*.

### Theoretical example

**Figure 1:**
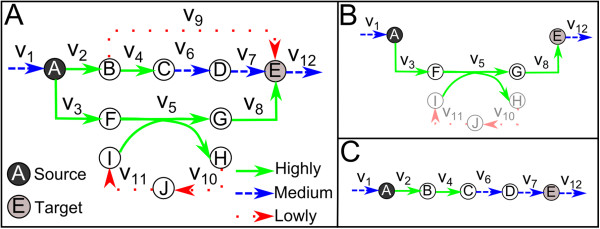
**Illustrative example. (A)** Metabolic network, **(B)** Pathway obtained when stoichiometry balance is neglected, **(C)** Pathway obtained with iCFP that accounts for stoichiometry balance. Highly/over expressed reactions are represented by solid lines, Medium expressed/invariant reactions by dashed lines and Lowly/down expressed reactions by dotted lines.

We explain our iCFP approach by the example presented in Figure 1A which involves 10 metabolites and 12 reactions. The source and target metabolites for the CFP are A and E, respectively. Assume that, based on expression data, reactions are classified as follows: *L* = {9,10,11}, *M* = {1,6,7,12} and *H* = {2,3,4,5,8}.

Classical path finding approaches, where stoichiometry is neglected, only take into account the reactions on the path. For the example shown in Figure 1A, the solution given by a classical approach, ignoring the reactions needed to balance the path, is shown in Figure 1B. Note how the path from A to E in Figure 1B is made up entirely from highly expressed reactions (reactions 3, 5, 8 in *H*). Even though this path is highly expressed, it needs flux in reactions 10 and 11 to achieve stoichiometric balance. This fact diminishes the relevance of the path since reactions 10 and 11 are lowly expressed. This demonstrates precisely how off-path reactions may alter the pathway enrichment structure.

On the contrary, when iCFP is applied (so including stoichiometric balance), the solution is as shown in Figure 1C. Note that the solution utilizes a mix of reactions from *H* (reactions 2 and 4) and *M* (reactions 6 and 7), but no reactions from *L* when balancing the path.

By means of this toy example, we show that iCFP, in contrast to classical approaches in the literature, obtains relevant paths with a consistent stoichiometric balance according to expression data. This theoretical example is now extended to a real metabolic scenario.

### Validation

In this subsection, using a more realistic scenario, we reinforce the importance of considering stoichoimetric balance when selecting relevant paths based on expression data. In particular, we analyze *L-Alanine* (L-Ala) degradation pathways to *Pyruvate* (Pyr) during biofilm formation of a genetically modified strain of *E. coli*.

**Figure 2:**
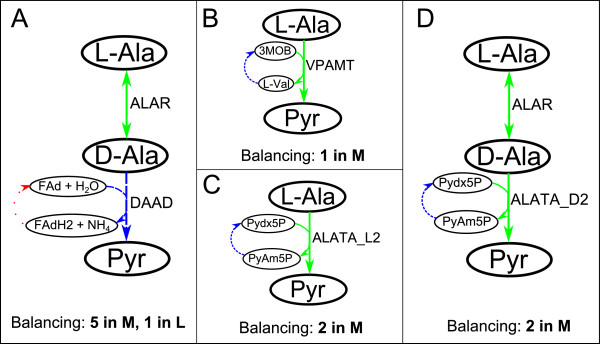
**Pathways for L-Alanine degradation to Pyruvate. A** Canonical pathway extracted from [53]; **B**, **C**, **D** Alternative pathways calculated using iCFP. Metabolites: 3MOB, 3-Methyl-2-oxobutanoate; D-Ala, D-Alanine; L-Val, L-Valine; NH_4_, Ammonium; Pydx5P, Pyridoxal 5′-phosphate; PyAm5P, Pyridoxamine 5′-phosphate. Reactions: ALAR, alanine racemase; ALATA_D2, D-alanine transaminase; ALATA_L2, alanine transaminase; DAAD, D-Amino acid dehydrogenase; VPAMT, Valine-pyruvate aminotransferase. Highly/over expressed reactions are represented by solid lines, Medium expressed/invariant reactions by dashed lines and Lowly/down expressed reactions by dotted lines.

Details as to the reactions constituting the backbone of L-Ala degradation pathway can be found in EcoCyc [53], which are summarized here in Figure 2A. Note that the last step of this pathway, catalyzed by *D-Amino acid dehydrogenase* (DAAD), consumes and produces *Flavin adenine dinucleotide oxidized* (FAD) and *Flavin adenine dinucleotide reduced* (FADH2), respectively. We show below that the balancing of these two compounds plays a crucial role, confirming that the example presented in the previous section goes beyond a hypothetical scenario.

We took data from [54]. Kim and co-workers provide gene expression data after modifying a particular set of genes related to biofilm formation in *E. coli* (GSE14203). Several gene expression comparisons between cultures are presented in terms of log_2_ fold changes. In particular, we focus on the comparison between wild type and *mqsR* mutant *E. coli* (GSM355066/GSM355065). As in [55], those genes with a fold change above 1.5 (log_2_ (fold change) > 0.5850) are considered highly/over expressed genes; on the contrary, genes with a fold change below -1.5 (log_2_ (fold change) < -0.5850) are included within the set of lowly/down expressed genes. If a gene is neither highly nor lowly expressed, it is classified as a medium expressed gene.

We incorporate gene expression data into the genome-scale metabolic network of *E. coli* in [51]. Following the logic rules relating gene/protein expression data with the final enzyme activity presented in [51], we obtain the final reaction classification into sets *H*, *M* and *L*. We used the same growth medium (Lysogeny Broth (LB)) in our simulations as in [54].

With the conditions described above, when iCFP is applied between L-Ala and Pyr, three paths are obtained (Figure 2B, C, D) before recovering the canonical solution (Figure 2A). Despite the similarity in the backbone of the pathways, the balancing produces a remarkable difference in terms of number of reactions in *L* and *M*, especially between the first three (Figure 2B, C, D) and the fourth (Figure 2A) pathway. Observe, for instance, that we obtained the same intermediates in the canonical pathway in Figure 2A and the third solution in Figure 2D. However, the set of co-substrates and by-products for each pathway is different, namely *Pyridoxal 5′-phosphate* (Pydx5P) and *Pyridoxamine 5′-phosphate* (PyAm5P) for Figure 2D, while FAD and FADH2 for Figure 2A. We found that the balancing of FAD and FADH2 requires the activation of at least one reaction in *L*, while for Pydx5P and PyAm5P only reactions in *M* and *H* are required. For this reason the pathway in Figure 2D was ranked in a better position than the canonical pathway in Figure 2A.

As a technical note, consider that FAD/FADH2 is tightly bound to a protein, in our case to *D-amino acid dehydrogenase* (*DAAD)* in Figure 2A. Because of this, the reaction cannot be balanced by any arbitrary FAD-reducing reaction in the network, but only by those that do not require FAD to be bound by a different protein. This lack of differentiation is a limitation of the employed metabolic network but not of the proposed method. Without taking into account this differentiation, we found that the stoichiometric balance of the canonical pathway in Figure 2A requires at least five and one enzymes in *M* and *L*, respectively. If this differentiation had been considered, we can only expect a worse (or equal) position in the ranking for the canonical pathway in Figure 2A, which reinforces the need for alternative pathways. Solutions obtained in Figure 2B, C, D do not include FAD/FADH2 and, hence, this issue does not apply here.

In summary, with this example of L-Ala degradation pathways into Pyr, we show that iCFP successfully accounts for stoichiometric balancing when selecting pathways based on gene expression data. This constitutes a progress over the state of the art, as it is typically neglected in classical path finding methods.

### Case study: acetate overflow metabolism in E. coli

Recently, Valgepea et al., 2010 [43] studied the specific growth rate-dependent metabolism of *E. coli* from a systems biology viewpoint using advanced steady-state continuous cultivation (A-stat [56]) by integrating genome-wide metabolomics, transcriptomics and proteomics measurements. In that work, a novel regulation mechanism for acetate overflow was elucidated. In particular, they propose that acetate overflow metabolism in *E. coli* is triggered by the disruption of the PTA-ACS node, namely *acetyl-CoA synthetase* (ACS) down-regulation results in decreased assimilation of *acetate* via *phosphotransacetylase* (PTA). We apply iCFP using the gene and protein expression data from [43] and the metabolic network of *E. coli* presented in [51] to gain further insights into key and novel pathways involved in acetate overflow metabolism. The criterion to classify reactions into *L*/*M*/*H* sets is included in the Methods section.

Since the consequence of acetate overflow metabolism is the excretion of *Acetate* (Ac) [44], we set Ac as the target metabolite with *Glucose* (D-Glc), the unique external input of carbon in the experimental growth medium in [43], as the source metabolite. We applied iCFP to this scenario and found 100 paths from D-Glc to Ac (Additional file 1). As an indication of the effect of incorporating gene and protein expression data, we also calculated here 100 paths from D-Glc to Ac ignoring expression data (*i.e.* ignoring the first two stages in iCFP). Only 10 of 100 paths were the same in both scenarios, namely with and without gene expression data. In other words, our results here indicate that incorporating expression data in the manner described above does significantly alter the set of paths found. A detailed analysis that discusses the effect of incorporating expression data and the resulting paths can be found in Additional file 2.

Of the 100 paths from D-Glc to Ac determined using iCFP, we observed that 98 had *Acetyl Coenzyme A* (AcCoA) as an intermediate metabolite. This is in line with a previous hypothesis for acetate overflow metabolism [57,58], which suggests that AcCoA diverts from entering the TCA cycle into formation of Ac. In order to capture the diversity in Ac producing pathways from AcCoA, we then calculated 100 paths setting AcCoA as the source metabolite and Ac as the target metabolite. We discuss and summarize below the most relevant pathways obtained via iCFP in this new scenario. The full set of pathways is included in Additional file 1.

**Figure 3:**
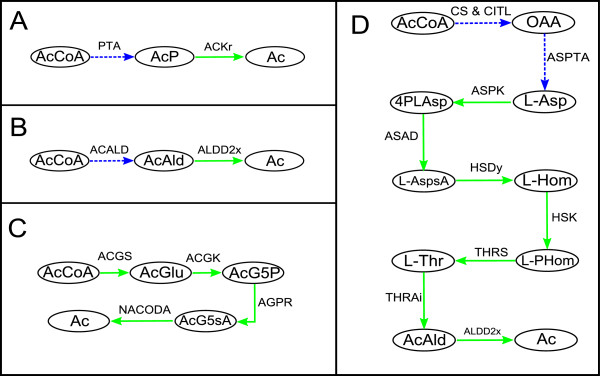
**Four relevant paths for acetate overflow calculated by iCFP. A**, First path; **B**, Second path; **C**, Third path; **D**, Fourth path. Highly/over expressed reactions are represented by solid lines, Medium expressed/invariant reactions by dashed lines and Lowly/down expressed reactions by dotted lines. Metabolites: Ac, Acetate; AcAld, Acetaldehyde; AcCoA, Acetyl-CoA; AcG5P, N-Acetyl-L-glutamyl 5-phosphate; AcG5sA, N-Acetyl-L-glutamate 5-semialdehyde; AcGlu, N-Acetyl-L-glutamate; AcP, Acetyl phosphate; D-Glc, D-Glucose; L-Asp, L-Aspartate; L-AspsA, L-Aspartate 4-semialdehyde; L-Hom, L-Homoserine; L-PHom, O-Phospho-L-homoserine; L-Thr, L-Threonine; OAA, Oxaloacetate; Pyr, Pyruvate. Reactions: ACALD, acetaldehyde dehydrogenase (acetylating); ACGK, acetylglutamate kinase; ACGS, N-acetylglutamate synthase; ACKr, acetate kinase; AGPR, N-acetyl-g-glutamyl-phosphate reductase; ALDD2x, aldehyde dehydrogenase (acetaldehyde, NAD); ASAD, aspartate-semialdehyde dehydrogenase; ASPK, aspartate kinase; ASPTA, aspartate transaminase; HSDy, homoserine dehydrogenase (NADPH); HSK, homoserine kinase; NACODA, N-acetylornithine deacetylase; PDH, pyruvate dehydrogenase; PTA, phosphotransacetylase; THRAi, Threonine aldolase; THRS, threonine synthase.

The first path appearing in the ranking is one half of the PTA-ACS cycle recently proposed by Valgepea et al., [43] as being central in the regulation of acetate metabolism (see Figure 3A). In essence, this pathway produces Ac from AcCoA with *Acetyl phosphate* (AcP) as an intermediate metabolite. In particular, AcP is transformed to Ac through the highly expressed reaction *acetate kinase* (ACKr). Further details regarding this mechanism can be found in Valgepea et al., [43].

A very similar pathway is obtained in the second position (Figure 3B). Note that this second solution is equivalent to the first pathway in terms of the optimization criterion using gene and protein expression data. In analogy with the mechanism presented in Valgepea et al., [43], this is a two-step procedure from AcCoA to Ac, but through a different intermediate metabolite *Acetaldehyde* (AcAld). In addition, note that the enzyme producing Ac from AcAld (*aldehyde dehydrogenase* (ALDD2x)) is over-expressed (Figure 3B). It is worth mentioning that the close distance between AcCoA and Ac makes this pathway a promising target to impair acetate overflow. Since the possible role of AcAld in acetate overflow metabolism has not been previously studied in depth, further experimental research is required to validate this hypothesis.

**Figure 4:**
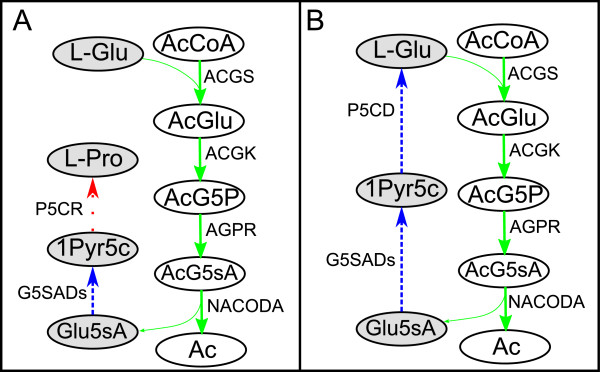
**A, Glu5sA is consumed to produce L-Pro; B, Glu5sA is consumed to produce L-Glu.** Highly/over expressed reactions are represented by solid lines, Medium expressed/invariant reactions by dashed lines and Lowly/down expressed reactions by dotted lines. Metabolites: 1Pyr5c, 1-Pyrroline-5-carboxylate; Ac, Acetate; AcCoA, Acetyl-CoA; AcG5P, N-Acetyl-L-glutamyl 5-phosphate; AcG5sA, N-Acetyl-L-glutamate 5-semialdehyde; AcGlu, N-Acetyl-L-glutamate; Glu5sA, L-Glutamate 5-semialdehyde; L-Glu, L-Glutamate; L-Pro, L-Proline. Reactions: ACGK, acetylglutamate kinase; ACGS, N-acetylglutamate synthase; AGPR, N-acetyl-g-glutamyl-phosphate reductase; G5SADs, L-glutamate 5-semialdehyde dehydratase (spontaneous); NACODA, N-acetylornithine deacetylase; P5CD, 1-pyrroline-5-carboxylate dehydrogenase; P5CR, pyrroline-5-carboxylate reductase.

iCFP also provides the pathway in Figure 3C within the first five solutions. This pathway comprises part of Arginine and Proline metabolism [34] with all the reactions between AcCoA and Ac catalyzed by highly-expressed enzymes *i.e.* all reactions included in *H*. Note that this pathway (Figure 3C) consumes and produces a molecule of *L-Glutamate* (L-Glu) and *L-Glutamate 5-semialdehyde* (Glu5sA), respectively. Since extracellular accumulation of Glu5sA was not detected in these experiments, we will analyze two mechanisms to balance this by-product:

1. On one hand, Glu5sA can be consumed to produce *Proline* (L-Pro) in a two-reaction pathway (*L-glutamate 5-semialdehyde dehydratase (spontaneous)* (G5SADs) and *pyrroline-5-carboxylate reductase* (P5CR)). This pathway represents the canonical biosynthetic mechanisms of L-Pro [59]. This is summarized in Figure 4A.

2. On the other hand, by means of another two-enzyme pathway constituted by G5SADs and *1-pyrroline-5-carboxylate dehydrogenase* (P5CD), Glu5sA can be consumed to produce the required molecule of L-Glu consumed in ACGS reaction so that the pathway in Figure 3C can be balanced, see Figure 4B. This is precisely the solution provided by iCFP.

In essence, both paths differ in the last enzyme *i.e.* P5CR for L-Pro production and P5CD in the case of L-Glu, respectively. Interestingly, P5CR is classified as belonging to the set *L*, while P5CD is classified as belonging to the set *M* (Figure 4). We can conclude that the second pathway consuming Glu5sA is more favorable based on gene and protein expression data (Figure 4B). This may imply that L-Pro, an essential amino acid for cellular proliferation, is not a limiting resource at faster growth, in line with preliminary experiments conducted in [60,61]. In addition, the second pathway seems to more efficient for acetate production, as L-Glu is balanced and therefore carbon is not diverted into other by-products, such as L-Pro.

Finally, we discuss the pathway in Figure 3D. AcCoa is first consumed to produce *Oxaloacetate* (OAA). Then, OAA is degraded by means of *L-Threonine* (L-Thr) metabolism [34] so that Ac is finally produced. Note that the first step is constituted by the action of two enzymes, namely *citrate synthase* (CS) and *citrate lyase* (CITL). It is important to note that there is no effective carbon exchange between AcCoA and OAA through these two reactions. In contrast, it can be verified that Ac is fully composed by carbon atoms from OAA. As OAA can be produced by several mechanisms, such as *glycolysis,* effective carbon exchange is guaranteed between D-Glc and OAA, and in consequence between D-Glc and Ac. In this light, OAA can be produced from the glycolytic product *phosphoenolpyruvate* (PEP) by *phosphoenolpyruvate carboxylase* (PPC), which is classified as belonging to the set *H*.

We are aware that this pathway is not particularly efficient for Ac production, as it requires 10 enzymatic steps and the consumption of 2 molecules of ATP and NADPH, respectively. However, this long path involves a well-known route for the biosynthesis of L-Thr, an essential amino acid for biomass synthesis. In addition, in the reaction catalyzed by *Threonine aldolase* (*THRAi*), L-Thr is degraded into glycine (Gly) and AcAld, which is then converted into Ac via *aldehyde dehydrogenase* (ALDD2x). Given that this pathway was obtained as differentially (highly) expressed with increasing specific growth rate, Gly and L-Thr should be synthesized at a high rate and, therefore, this pathway is likely to occur also at a high rate. The use of less efficient pathways also emphasizes increasing carbon wasting with faster growth as experimentally observed (mainly associated with acetate overflow). It is interesting to note in the context of acetate overflow that higher biomass yields are observed by supplementing minimal medium with Gly and L-Thr in addition to glucose [60], which is in line with the results presented above, where these two amino acids appear to be a limiting resource.

## Conclusions

There is a large volume of gene expression data available through different public databases. In addition, due to the rapid advancement in proteomics technologies, protein abundance data is increasing day by day, as well as absolute quantitative –omics data. In order to exploit this valuable information, we require models and efficient algorithms to extract biological conclusions.

Genome scale networks have shown that cellular metabolism underlies a wide number of phenotypes. As discussed elsewhere [32], representing those phenotypes by means of the well-known canonical metabolic pathways, may be a limited strategy. Thus, it is essential to use mathematical pathway models which allow us to calculate more solutions, going beyond canonical metabolic pathways. Amongst pathway models, the Carbon Flux Path model appears as a promising tool that extends classical path finding techniques by incorporating biophysical constraints, such as mass balance. In this article, we emphasized the relevance of including these biophysical constraints (stoichiometric balance) by means of, firstly, a theoretical example and, secondly, the analysis of L-Ala degradation pathways into Pyr during biofilm formation of a genetically modified strain of *E. coli*. In order to take into account the balancing in the obtained paths, the use of MILP is required. Efficient MILP solvers exist and therefore the application of iCFP to genome-scale metabolic networks is feasible, as shown in the Results section.

As posed above, genome scale metabolic networks may present assorted phenotypes. In order to correctly represent those phenotypes, it may be necessary to pre-calculate an extremely large number of metabolic pathways. The framework presented here overcomes this issue by directly calculating the best metabolic pathways under a given gene/protein expression phenotype. The effect of gene and protein expression data in our approach is clearly observed, particularly leading to two significantly different sets of pathways depending upon whether this information is included or not.

In iCFP, reactions are classified into *H*, *M* or *L* sets. We defined these sets based on a standard procedure (see Methods section). However, this problem deserves further research, as complex effects may arise. For instance, reducing the concentration of a particular protein may not lead to a proportional decrease in its catalytic activity [62,63]. Therefore, other factors, such as thermodynamic properties, dynamics of catalytic rates of enzymes, etc, should be included to more accurately classify reactions.

Based on the above, we proposed novel metabolic pathways involved in acetate overflow metabolism, which might be targets to possibly mitigate acetate overflow and help to understand the regulation of this phenomenon. The role of *Acetaldehyde* is of particular interest, which to our knowledge has not been previously related to acetate overflow. In addition, we also discussed two other over-expressed metabolic pathways possibly relevant for acetate overflow. The evidence of acetate production directly linked to Gly and L-Thr seems plausible, as their importance for increasing biomass yield per substrate has been previously reported and explored in the context of acetate overflow. We are aware that overflow metabolism is a more general problem and additional studies, which complement the one presented here, need to be carried out to design an optimized strain, as if acetate secretion is blocked, the strain may still export other compounds such as lactate at a high rate.

As future lines for research into acetate overflow, note that our insights were obtained from the metabolic model presented in [51], as carbon exchange arcs associated with reactions, which constitute a key input for the CFP approach, are currently only available for this model. Developing a database of carbon exchange arcs for the most recent *E. coli* genome-scale metabolic network [64] would need to be done so as to verify and complement obtained results. Additionally, isotope labeling methods would be beneficial to validate the proposed pathways obtained by iCFP. Finally, as one of our analyzed pathways involves the well-known signal molecule AcP, which plays a critical role in regulation of chemotaxis, pathogenesis, biofilm formation etc. [43] and protein acetylation [65], it seems relevant to try and detect a relationship between signal transduction pathways and metabolic alterations associated with acetate overflow.

In conclusion, the increasing efficiency of high-throughput technologies in association with novel computational frameworks lays the foundations for future discoveries. Overall, methods exploiting -omics data, as is the case of iCFP presented in this article, are essential to unravel the complex biological scenarios that exist in different areas of health science and biotechnology. *E. coli* acetate overflow is one of these complex scenarios. With our iCFP approach, we were able to develop non-trivial insights that contribute to the better understanding of this phenomenon relevant for industry biotechnology.

## Methods

### Classifying reactions into H, M and L sets based on specific growth rate-dependent gene and protein expression data

**Figure 5:**
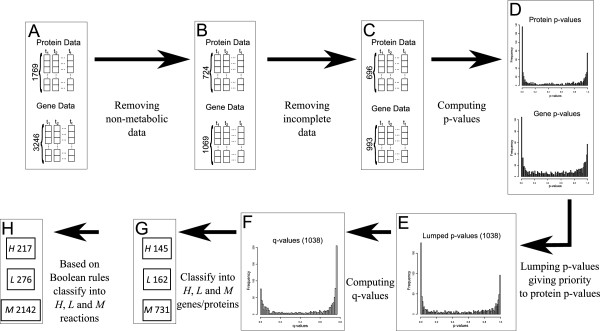
**Flowchart showing the procedure to classify genes as up and down regulated from the data of Valgepea et al. **[43]**. A**,**B**,**C**,**D**,**E**,**F**,**G**,**H** Individual steps in the procedure.

The data presented in [43] contains experimental measurements of gene and protein expression at different specific growth rates (Figure 5A). Due to the regulatory mechanisms between gene expression and protein production, proteomic data is more reliable when inferring metabolic phenotypes. However, more measurements as to gene expression data are available. Thus, when proteomic data was not available or incomplete, we used gene expression data. When neither gene nor protein expression data was provided for a particular gene, we assume that the associated gene is invariant over the studied range of specific growth rates. Further details are included below.

In order to properly classify as up and down regulated genes/proteins, the data provided by Valgepea et al., [43] is normalized by applying logarithm to the base 2 (Log_2_), which is particularly common in the literature [66]. Those genes/proteins not involved in the *E. coli* metabolic networks are removed (Figure 5B). Then, those genes and proteins with three or less measurements over the studied range were removed from the analysis and directly included in the set of non-differentially expressed features (Figure 5C).

Genes or proteins may show random behavior without a clear indication of either down regulation or up regulation. It is crucial to distinguish between genes/proteins with and without regular change of expression over the course of study; this can be done via linear regression. This technique is efficiently implemented in the limma package [67]. In particular, the treat function [68] included in limma provides the p-value of having an up or down regulation more extreme than a selected threshold. The corresponding null hypothesis for this test is that the gene is up or downregulated less than the threshold*, T*. As in previous works [69,70], this pre-defined threshold is set to 1.5-fold changes, *i.e.* the (ratio) change in expression between the initial and final condition increases or decreases by 50%. Note that these probabilities are calculated for gene and protein expression data and, therefore, two sets of p-values are calculated (Figure 5D). We conducted a sensitivity analysis for different *T* values. Results can be found in Additional file 2.

Our two sets of p-values included in Figure 5D are combined into a unique set. For those enzymes with two associated p-values, namely from the protein and gene expression data, the p-value corresponding to the proteomics data is assigned. When the p-value calculated with proteomics data is not available, the p-value obtained using gene expression data is assigned. Finally, when neither protein nor gene p-value is available, the gene is directly included in the set of non-differentially expressed. After grouping the p-values, a unique set of 1038 values is obtained (Figure 5E).

In addition, since this analysis implies multiple independent tests, we have corrected the obtained p-values so as to obtain the set of q-values. This correction is performed using fdrtool [71] implemented in the R language. fdrtool estimates the False Discovery Rate (FDR) for a set of independent p-values. After multiple hypothesis correction, the genes with FDR below 0.2 (20%), are considered to be differentially expressed [72,73]. Using this FDR, we found 307 metabolic genes differentially expressed (Figure 5F). Note that the set of differentially expressed genes/proteins are classified as up or down regulated if the sign of the regressed curve provided by limma is positive or negative respectively. This resulted in 145 up regulated and 162 down regulated genes/proteins (Figure 5G).

Finally, following the strategy adopted in previous works [48], we classify all the metabolic reactions in the set of the Highly/Over expressed reactions (*H*), the set corresponding to the Lowly/Down expressed (*L*) and the set of Medium expressed/Invariant reactions (*M*) based on the Boolean rules included in [51]. Overall, 217, 276 and 2142 reactions are included in the *H*, *L* and *M* sets, respectively.

### Carbon flux paths

Drawing directly on [38], the model for carbon flux paths is as follows. *C* contains all the metabolites present in the metabolic network whilst *R* contains all the reactions. Reversible reactions contribute two different reactions to the metabolic network, so all fluxes (flux *v*_*r*_ for reaction *r*) take positive values. *S*_*cr*_ is the stoichiometric coefficient associated with metabolite *c* in reaction *r*. The source and target metabolites are *α* and *β*, respectively. A flux path is a simple path from *α* to *β* able to operate in steady-state. Arcs in the path are given by the zero-one (binary) variable *u*_*ij*_ = 1 if the arc *i*→*j* linking metabolite *i* to metabolite *j* is active in the path, 0 otherwise. The zero-one (binary) variable *z*_*r*_ is equal to 1 if reaction *r* has non-zero flux, 0 otherwise. The coefficient *d*_*ijr*_ is one if there exists effective carbon exchange between input metabolite *i* (*S*_*ir*_ < 0) and output metabolite *j* (*S*_*jr*_ > 0) in reaction *r* (and is zero otherwise). The constraints in the model are:

(1) $$\sum_{j\in C}u_{\alpha j}=\sum_{i\in C}u_{i\beta }=1$$

(2) $$\sum_{i\in C}u_{i\alpha }=\sum_{j\in C}u_{\beta j}=0$$

(3) $$\sum_{i\in C}u_{\mathrm{ik}}=\sum_{j\in C}u_{\mathrm{kj}}\forall k\in C;k\neq \alpha ,\beta$$

(4) $$\sum_{i\in C}u_{\mathrm{ik}}\leq 1\;\forall k\in C$$

(5) $$\sum_{r\in R}S_{\mathrm{cr}}v_{r}=0\;\forall c\in I$$

(6) $$\sum_{r\in R}S_{\mathrm{cr}}v_{r}\geq 0\;\forall c\in E,c\notin E_{m}$$

(7) $$z_{r}\leq v_{r}\leq Nz_{r}\;\forall r\in R$$

(8) $$z_{\lambda }+z_{\mu }\leq 1\;\forall \left(\lambda ,\mu \right)\in B$$

(9) $$\sum_{r\in R,d_{\mathrm{ijr}}=1}z_{r}\geq u_{\mathrm{ij}}\;\forall i\in C;\forall j\in C;i\neq j$$

Equation (1) ensures that one arc leaves *α* and one arc enters *β*. Equation (2) that no arc enters α and no arc leaves *β*. Equation (3) ensures that the number of arcs entering a metabolite *k* is equal to the number leaving. Equation (4) ensures that a metabolite cannot be revisited in the path. Equation (5) is the steady-state condition for the set (*I*) of internal metabolites. Equation (6) ensures that metabolites not involved in a specific growth medium (*E*_*m*_) can be produced, but cannot be consumed. Equation (7) relates each flux with a binary variable that indicate whether the reaction is active or not, where *N* is the maximum flux and the minimum (non-zero) flux is 1. Equation (8) prevents a reaction and its reverse both being active where *B* = {(*λ,μ*)| reaction *λ* and reaction *μ* are the reverse of each other}. Equation (9) ensures that if a carbon exchange arc is used in the path, then some reaction involving such arc is active.

### Carbon flux paths accounting for expression data

With expression data, the set *R* of reactions has been subdivided into three mutually exclusive sets *L*/*M*/*H* of lowly/medium/highly expressed reactions. Our model is a three-stage optimization model. In the first-stage optimization, we minimize the total flux associated with lowly expressed reactions, namely those included in the set *L*:

(10) $$\text{Minimize}\;\sum_{r\in L}v_{r}$$

Subject to Equations (1)-(9)

This is a mixed-integer linear problem and algorithmically such problems are solved by linear programming based tree search. Modern software packages to perform this task, such as IBM ILOG CPLEX which we used, are well developed and highly sophisticated. IBM ILOG CPLEX was run in a Matlab environment Version 7.5 (R2007b).

If the optimal objective function value is *V*_*1*_, then, in the second-stage optimization, we minimize the total flux associated to reactions in *M, i.e.* medium expressed reactions:

(11) $$\text{Minimize}\;\sum_{r\in M}v_{r}$$

Subject to Equations (1)-(9) and

$$\sum_{r\in L}v_{r}=V_{1}$$

This ensures that we retain the optimal value (*V*_*1*_) for $\sum_{r\in L}v_{r}$ achieved at the first-stage.

If the optimal value here is *V*_*2*_, then, in the third-stage optimization, we minimize the length of the flux path from source to target metabolite:

(12) $$\text{Minimize}\;\sum_{i\in C}\sum_{j\in C,j\neq i}u_{\mathrm{ij}}$$

Subject to Equations (1)-(9) and $\sum_{r\in L}v_{r}=V_{1}$ and

$$\sum_{r\in M}v_{r}=V_{2}$$

This ensures that we retain the optimal values achieved at the first two stages.

Note here that the three stage optimization model can be converted into a single step optimization by introducing suitable weighting factors for each term in equations (10)-(12), namely very large for the first objective, less large for the second, etc. However, this introduces numerical issues as the different terms in the objective vary widely in value. So, for the sake of numerical stability, we opted for the three step optimization model.

Since the last stage here is to minimize the number of reactions, we can enumerate path solutions in ascending (staged) objective value order, so first find the CFP that best minimizes our three-stage objective; then the next best solution; then the next best solution; etc. To do this, after finding a path solution, we simply add a constraint eliminating it from the solution space. If *U*_*ij*_^*k*^ is the binary solution value for the *u*_*ij*_ variable in the *k*-shortest path (and we have *K* such paths) then the elimination constraint is:

(13) $$\sum_{i\in C}\sum_{j\in C,j\neq i}U_{\mathrm{ij}}^{k}u_{\mathrm{ij}}\leq \sum_{i\in C}\sum_{j\in C,j\neq i}U_{\mathrm{ij}}^{k}-1\;k=1,\ldots ,K$$

## Abbreviations

1Pyr5c: 1-Pyrroline-5-carboxylate; 3MOB: 3-Methyl-2-oxobutanoate; ACALD: Acetaldehyde dehydrogenase (acetylating); ACGK: Acetylglutamate kinase; ACGS: N-acetylglutamate synthase; ACKr: Acetate kinase; AGPR: N-acetyl-g-glutamyl-phosphate reductase; ALAR: Alanine racemase; ALATA_D2: D-alanine transaminase; ALATA_L2: Alanine transaminase; ALDD2x: Aldehyde dehydrogenase (acetaldehyde NAD); ASAD: Aspartate-semialdehyde dehydrogenase; ASPK: Aspartate kinase; ASPTA: Aspartate transaminase; Ac: Acetate; AcAld: Acetaldehyde; AcCoA: Acetyl-CoA; AcG5P: N-Acetyl-L-glutamyl 5-phosphate; AcG5sA: N-Acetyl-L-glutamate 5-semialdehyde; AcGlu: N-Acetyl-L-glutamate; AcP: Acetyl phosphate; CFP: Carbon Flux Path; CITL: Citrate lyase; D-Ala: D-Alanine; D-Glc: D-Glucose; DAAD: D-Amino acid dehydrogenase; EFM: Elementary Flux Mode; FAD: Flavin adenine dinucleotide oxidized; FADH2: Flavin adenine dinucleotide reduced; FDR: False discovery rate; G5SADs: L-glutamate 5-semialdehyde dehydratase (spontaneous); Glu5sA: L-Glutamate 5-semialdehyde; Gly: Glycine; HSDy: Homoserine dehydrogenase (NADPH); HSK: Homoserine kinase; L-Ala: L-Alanine; L-Asp: L-Aspartate; L-AspsA: L-Aspartate 4-semialdehyde; L-Glu: L-Glutamate; L-Hom: L-Homoserine; L-PHom: O-Phospho-L-homoserine; L-Pro: L-Proline; L-Thr: L-Threonine; L-Val: L-Valine; LB: Lysogeny Broth; MILP: Mixed-Integer Linear Programming; NACODA: N-acetylornithine deacetylase; NH4: Ammonium; OAA: Oxaloacetate; P5CD: 1-pyrroline-5-carboxylate dehydrogenase; P5CR: Pyrroline-5-carboxylate reductase; PDH: Pyruvate dehydrogenase; PEP: Phosphoenolpyruvate; PPC: Phosphoenolpyruvate carboxylase; PTA: Phosphotransacetylase; PyAm5P: Pyridoxamine 5′-phosphate; Pydx5P: Pyridoxal 5′-phosphate; Pyr: Pyruvate; THRAi: Threonine aldolase; THRS: Threonine synthase; VPAMT: Valine-pyruvate aminotransferase; iCFP: Integrative carbon flux path.

## Competing interests

The authors declare that they have no competing interests.

## Authors’ contributions

JP conceived, developed and implemented the method, wrote the manuscript; KV developed the biological validation and wrote the manuscript; AR, designed the statistical analysis and wrote the manuscript; JEB developed the method and wrote the manuscript; FJP conceived the study, developed the method and wrote the manuscript. All authors discussed the results, and read, commented and approved the final manuscript.

## Supplementary Material

Additional file 1 Details for pathways between Glucose-Acetate and Acetyl Coenzyme A-Acetate. Click here for file

Additional file 2 **Effect of gene expression data and sensitivity analysis on *T *** **value.** Click here for file
