# Supplementary material for: Integrating gene and protein expression data with genome-scale metabolic networks to infer functional pathways
Source: BMC Syst Biol. 2013 Dec 8;7:134. doi: 10.1186/1752-0509-7-134 (PMC3878952; doi:10.1186/1752-0509-7-134)
Supplement: Additional file 2 — Effect of gene expression data and sensitivity analysis on T value. [file 1752-0509-7-134-S2.docx]

**Additional File II**

**Integrating gene and protein expression data with genome-scale metabolic networks to infer functional pathways**

Jon Pey^1^, Kaspar Valgepea^2, 3^, Angel Rubio^1^, John E. Beasley^*,4^ and Francisco J. Planes^*,1^

In this Supplementary Material, we present two additional studies in the context of the acetate overflow discussion in the main text. Firstly, we describe in more detail the impact of including (or not including) expression data into iCFP. Secondly, a sensitivity analysis of iCFP with respect to the threshold that defines the set of highly (*H*), medium (*M*) and lowly (*L*) expressed reactions is performed.

**Effect of expression data**

We present here an analysis of the number of highly (*H*), lowly (*L*) and medium (*M*) expressed reactions involved in the first 100 paths between D-Glc and Ac with, and without, gene-protein expression data. When gene expression data was not included, we assumed that all reactions were in the *M* set. In order to clarify the differences seen, a pie chart is presented in Figure S1.

Notable differences are observed. In particular, note how the number of lowly expressed reactions decreased to zero whilst the number of highly expressed reactions increased almost 4 times when expression data was introduced. This analysis clearly shows that including expression data has a significant effect on the set of pathways, particularly as reactions in *L* and *M* are penalized, meaning that reactions in *H* are preferred. For example, as noted in the main text, when expression data was used, we obtained 98 pathways using AcCoA as an intermediate. However, this number decreased to 44 when expression data was neglected and, therefore, the pathways produced are significantly different.


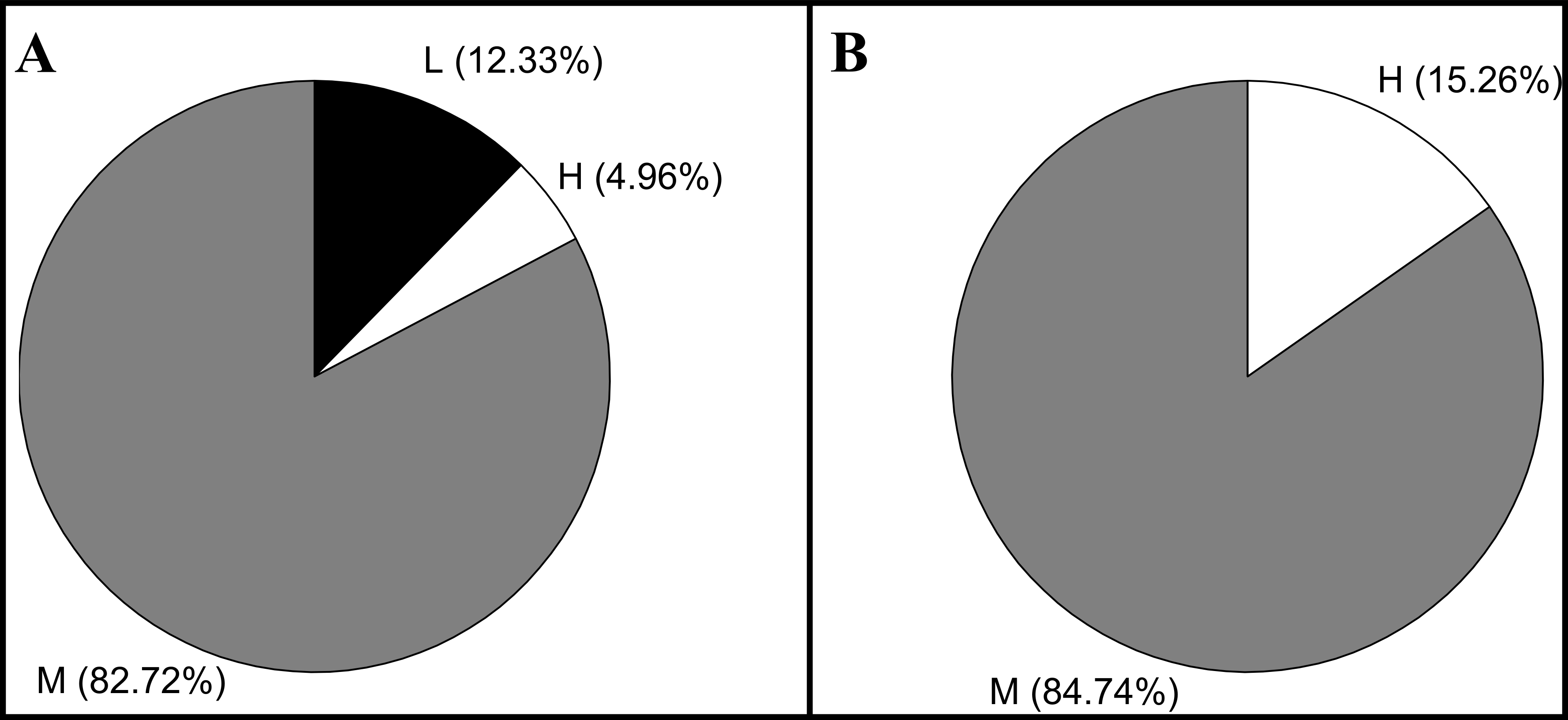


**Figure S1:** Percentage of reactions in *H*, *L* and *M* when expression data was (B) and was not (A) used

**Sensitivity Analysis**

As noted in the main text, in order to categorize genes as up-regulated and down-regulated, we used the *treat* function (McCarthy and Smyth, 2009) included in *limma* package (Smyth, 2005), which provides the p-value of having an up or down regulation more extreme than *T* fold changes. In particular, for the set of 100 paths from AcCoA to Ac discussed in the main text, we used *T*=1.5.

Here, we present a sensitivity analysis aiming to study the effect of *T* in the set of calculated pathways. We started our analysis from *T*=1. In particular, as *treat* function uses |log_2_ *T*|, we obtain the same result for *T*=2 and *T*=1/2; for *T*=3 and *T*=1/3, etc, and, therefore, an analysis of the interval [0,1) is not required. For simplicity, we varied *T* between 1 and 2 in intervals of length 0.01, increasing the interval size to 0.1 between 2 and 5. For each case, we calculated 100 pathways via iCFP and compared them with the solutions obtained for *T*=1.5, specifically the number of solutions (paths) common in both scenarios was calculated. Results can be found in Figure S2.

Firstly, it can be observed in Figure S2 that when *T*=1.5 similarity is 100, as this is the score used in the main paper. Solutions in that case are termed here the reference set. Secondly, for *T*>1.5, as *T* is more stringent, the *M* set is more populated and, therefore, the effect of expression is lessened and differences with respect to the reference set arise. This behavior becomes extreme for *T*>2.1, where the pathways obtained barely change, as the *H*, *L* and *M* sets remain constant. In addition, for *T*<1.5, as *T* is less stringent, more reactions will be involved in the *H* and *L* sets and, therefore, solutions will differ from the reference set, with an extreme situation for *T*=1, where only 4 of the 100 paths are common.

Finally, it is relevant to emphasize that iCFP shows a more robust behavior for *T* values around 1.5, obtaining a similar set of solutions, namely at least 80 of the 100 are common for 1.4<*T*<1.61. This robustness validates the biological relevance of results for *T*=1.5, the default value used in a number of experimental studies in the literature.


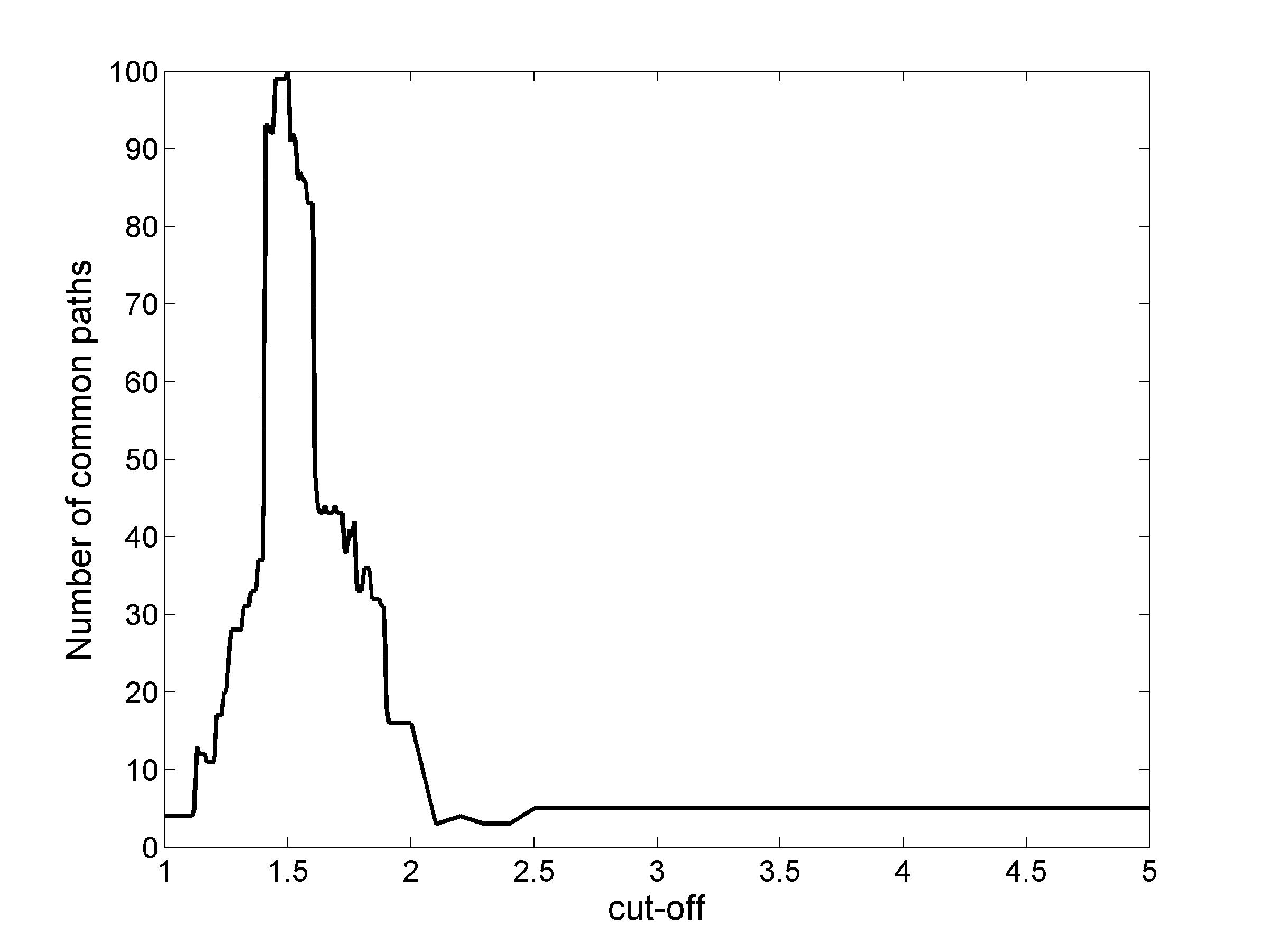


**Figure S2:** Sensitivity analysis of obtained paths for different *T* values

**References**

McCarthy DJ, Smyth GK: **Testing significance relative to a fold-change threshold is a TREAT**. *Bioinformatics* 2009, **25**:765–771.

Smyth G: **Limma: linear models for microarray data**. *Bioinformatics and computational biology solutions using R and Bioconductor* 2005:397–420.
